# Effect of resveratrol on mouse ovarian vitrification and transplantation

**DOI:** 10.1186/s12958-021-00735-y

**Published:** 2021-04-09

**Authors:** Dalin Wang, Menghui Geng, Dongying Gan, Gege Han, Gao Gao, Aying Xing, Yugui Cui, Yanqiu Hu

**Affiliations:** 1 State Key Laboratory of Reproductive Medicine, Clinical Center of Reproductive Medicine, First Affiliated Hospital, Nanjing Medical University, Nanjing, 210029 Jiangsu Province China; 2 Department of Obstetrics and Gynecology, Clinical Medical School of Yangzhou University, Yangzhou, 225000 Jiangsu Province China; 3 Department of Obstetrics and Gynecology, Dalian Medical University, Dalian, 116000 Liaoning Province China; 4 The kangda college of Nanjing medical university, Nanjing, 210029 Jiangsu Province China; 5 Reproductive Medicine Center of Northern Jiangsu People’s Hospital, Yangzhou University, Yangzhou, 225000 Jiangsu Province China

**Keywords:** Ovarian tissue, Transplantation, Vitrification, Resveratrol, Ischemic injury

## Abstract

**Background:**

After ovarian tissue transplantation, ischemia-reperfusion injury and free radicals cause follicle depletion and apoptosis. Therefore, the use of antioxidants to reduce the production of free radicals is an important method to address the consequences of ischemia-reperfusion injury. Resveratrol is a natural active polyphenol compound with anti-inflammatory, antitumor, strong antioxidant and anti-free radical properties. The aim of this study was to investigate whether resveratrol could improve the effect of autologous ovarian transplantation after cryopreserve-thawn mouse ovarian tissue.

**Methods:**

Whole-ovary vitrification and autotransplantation models were used to investigate the effects of resveratrol. Six-week-old female mice from the Institute of Cancer Research (ICR) were subjected to vitrification. All ovaries were preserved in liquid nitrogen for 1 week before being thawed. After thawing, ovarian tissues were autotransplanted in the bilateral kidney capsules. Mice (*n* = 72) were randomly divided into four groups to determine the optimal concentration of resveratrol (experiment I).

Treatments were given as follows: saline, 5 mg/kg resveratrol, 15 mg/kg resveratrol and 45 mg/kg resveratrol, which were administered orally for one week. Grafted ovaries were collected for analysis on days 3, 7, and 21 after transplantation. Ovarian follicle morphology was assessed by hematoxylin and eosin staining. Serum FSH and E_2_ levels were measured to estimate the transplanted ovarian reserve and endocrine function. Other mice were randomly divided into two groups—saline and 45 mg/kg resveratrol to further evaluate the effect of resveratrol and explore the mechanisms underlying this effect (experiment II). Ovarian follicle apoptosis was assessed by terminal deoxynucleotidyl transferase-mediated dUTP nick-end labeling (TUNEL) assays. Immunohistochemistry, qRT-PCR and western blotting (MDA, SOD, NF-κB, IL-6 and SIRT1) were used to explore the mechanisms of resveratrol. Moreover, oocytes derived from autotransplanted ovaries at 21 days were cultured and fertilized in vitro.

**Results:**

The proportions of morphologically normal (G1) follicles at 3, 7 and 21 days were significantly higher in the 45 mg/kg resveratrol group than in the saline group. The TUNEL-stained follicles (%) at 7 days were significantly decreased in the 45 mg/kg resveratrol group compared with the saline group. Western blot analysis revealed that SOD2 and SIRT1 levels were significantly higher in the 45 mg/kg resveratrol group than in the saline group at day 7 and that MDA and NF-κB levels were lower in the saline group on day 3. Likewise, IL-6 was lower in the saline group on day 7. These results are basically consistent with the qRT-PCR results. In addition, the mean number of retrieved oocytes and fertilization and cleavage were significantly increased in the 45 mg/kg resveratrol group compared with the saline group.

**Conclusions:**

Administration of resveratrol could improve the quality of cryopreserved mouse ovarian tissue after transplantation and the embryo outcome, through anti-inflammatory and antioxidative mechanisms.

**Supplementary Information:**

The online version contains supplementary material available at 10.1186/s12958-021-00735-y.

## Background

Diagnostic and treatment methods for cancer have dramatically improved over the past decades. However, many survivors develop early menopause, premature ovarian failure and infertility because of chemotherapy and radiotherapy [1, 2]. As the number of cancer patients increases, fertility preservation has become increasingly important to improve survivor quality of life and preserve fertility [3]. Currently, ovarian tissue (OT) cryopreservation and transplantation [4] represent the most effective option for fertility preservation for children, adolescents or women who cannot postpone chemotherapy and radiotherapy [5]. Many studies [2, 6] have indicated that OT cryopreservation and transplantation can improve fertility preservation and restoration. This strategy has resulted in more than 200 live births [7, 8]. Unfortunately, in OT transplantation without surgical vascular anastomosis, more than 70% of primordial follicles [9] are lost due to early ischemia injury between transplantation and revascularization [10]. Moreover, early ischemia-reperfusion injury and free radical and oxidative damage can cause oocyte, granulosa cell and membrane lipid damage or apoptosis [11]. These changes will increase microvascular permeability and OT swelling and activate inflammatory responses through activation of adhesion molecules and enhanced cytokine production. Reactive oxygen species (ROS) directly cause oxidative damage to nucleic acids, proteins, lipids and other cellular macromolecules in ischemic tissues [12, 13]. Therefore, the use of antioxidants to reduce the production of free radicals can be considered an important strategy to address the consequences of ischemia-reperfusion injury after OT autotransplantation [14]. To date, several attempts have been made to improve follicle survival in grafted OT [15, 16]. Various antioxidants, such as vitamin E [17], N-acetylcysteine [18], and erythropoietin [19], have been shown to yield some improvement in follicle apoptosis and lipid peroxidation. However, no long-term follow-up of grafted human OT has been performed, and further optimization studies are required to improve tissue grafting.

Resveratrol (trans-3.5,40-trihydroxystilbene) is a natural active polyphenol compound produced by plants and is primarily found in red wine, peanuts and polydatin [20]. This compound has been reported to have a wide variety of pharmacological properties, such as anti-inflammatory, antitumor, strong antioxidant and anti-free radical activities [21]. In particular, resveratrol can enhance the endogenous antioxidant system of cells and the reactive oxygen species scavenging function [22]. Many pathological conditions resulting from ischemia–reperfusion injury was believed to be preventable by resveratrol, which can reduce IL-1, IL-6 and TNF-β levels by downregulating the expression of NF-κB. The ability of resveratrol to suppress inflammatory indicators could reduce ovaries damage against toxicity and ischemia-reperfusion. Moreover, resveratrol has several effects on reproductive physiology, including maintenance of the ovarian reserve [23, 24] and protection against aging-related infertility [25]. Likewise, resveratrol supported the growth of human ovarian follicles in an in vitro culture model [26].

Based on findings from previous studies, this study aimed to investigate the effects of resveratrol on ischemia–reperfusion injury after autologous ovarian transplantation and to evaluate its effects on the ovarian graft survival rate and function and follicular development and associated mechanisms.

## Methods

### Animals

A total of 176 female mice 6–8 weeks-old from Institute of Cancer Research (ICR) mice (Yangzhou University Comparative Medicine Centre) were housed under standard conditions of temperature (23 °C ± 2 °C), relative humidity (65% ± 5%), and a 12-h light/dark cycle in Table 1. Food and water were provided ad libitum. The experimental protocols and animal handling procedures were approved and monitored by the Institutional Animal Care and Use Committee (IACUC) of Yangzhou University (SYXK (SU)2017–0044).

**Table 1:** Schematic for the study design

	Methods	Day (after transplantation)	Saline (number of mice)	5 mg/kg (number of mice)	15 mg/kg (number of mice)	45 mg/kg (number of mice)
Fresh	6					
vitrification	6					
ExperimentI		3 d	6	6	6	6
	HE/E_2_/FSH	7 d	6	6	6	6
		21 d	6	6	6	6
ExperimentII	TUNEL/IHC	3 d	6			6
		7 d	6			6
	Western blot	3d	6			6
		7d	6			6
	RT-PCR	3d	8			8
		7d	8			8
	IVM/IVF	21d	6			6

*Experiment I* Evaluation of the best concentration of resveratrol, *Experiment II* Further evaluation of the effects of resveratrol and exploration of the mechanisms

### Ovarian tissue vitrification and thawing

Mice (*n* = 6) were anesthetized by intraperitoneal (IP) injection of a solution of ketamine (0.15 mg/g of body weight) and xylazine (0.016 mg/g of body weight). Both ovaries were removed and freed of fat. Ovaries were vitrified according to the protocols described by Youmetal [27].. Briefly, intact OT was serially exposed to equilibration solution (ES) for 10 min and vitrification solution (VS) for 5 min [ES composition: Dulbecco’s phosphate-buffered saline (DPBS) supplemented with 20% (v/v) fetal bovine serum (FBS; Sigma, USA), 7.5%(v/v) dimethylsulfoxide (DMSO; Sigma, USA) and 7.5% (v/v) ethylene glycol (EG; Sigma, USA); VS composition: D-PBS containing 20% FBS, 20% EG, 20% DMSO and 0.5 M sucrose (Sigma, USA)]. The ovaries were placed on gauze to remove the vitrification solution. Then, we placed the ovaries in a 1.5-ml cryovial (Corning, Mexico) and placed the cryovial in liquid nitrogen (LN2).

A week after vitrification, the vials were rapidly removed from LN2, incubated at room temperature for 10–20 s and warmed through stepwise dilution of sucrose at concentrations of 1.0, 0.5, 0.25, and 0 M in D-PBS medium supplemented with 20% FBS for 5 min each. In the fresh (*n* = 6) and vitrification-warmed control groups, the OT was immediately fixed in 4% paraformaldehyde after ovariectomy or freezing and thawing, respectively.

### Experiment I: evaluation of the best concentration of resveratrol

Autotransplantation was performed as described in Lee et al. [28]. After analgesia was provided to the mice, the dorsal fur was shaved, and the abdominal skin was sterilized with 70% (v/v) alcohol. A 1-cm incision was made on the skin, and both kidneys were externalized through the incision site. A small hole was created, vitrification-warmed ovaries from a mouse were placed inside the bilateral kidney capsules, and the incision and skin were closed and sutured. The mice were randomly divided into four groups according to the treatment: saline (*n* = 18); 5 mg/kg resveratrol (n = 18); 15 mg/kg resveratrol (*n* = 18) and 45 mg/mg resveratrol (n = 18). Substances were given orally for one week. Resveratrol was insoluble in water and dissolved in 1% sodium carboxymethyl cellulose solution. Sodium carboxymethyl cellulose was diluted in saline. 1 ml of different concentration of resveratrol was given by oral gavage daily. The same volume of saline was given by oral gavage in the sham control group by using the same scheme described earlier. Ovarian grafts were retrieved 3, 7 and 21 days after transplantation to assess the morphology and graft survival. Six mice per group were used for histology and serum E_2_ and FSH analyses to select the most effective dose of resveratrol. Based on previous studies [29], we selected days 3, 7 and 21 for evaluation of ovarian follicle development and function. After mice were placed in airtight containers and anesthetized to death with an overdose of isoflurane.

### Follicle classification and morphological analysis

OTs were fixed in 4% paraformaldehyde and embedded in paraffin. The specimens were cut into 5-μm sections for morphological analysis or immunohistochemical staining. The sections were collected and stained with hematoxylin and eosin (H&E, Weiwo, China). Ovarian follicles were classified according to Lundy et al. [30] for developmental stage. The stages were defined as follows:

Primordial follicles: a single layer of flattened granulosa;

Primary follicles: a complete single layer of granulosa cells, one or more of which is cuboidal;

Secondary follicles: two or more layers of cuboidal granulosa cells with no antrum; Antral follicles: multiple layers of cuboidal granulosa cells with the antrum present.

Gandolfi’s [6] criteria were used for morphological integrity.

Primordial/primary follicle: G1, spherical with an even distribution of granulosa cells; G2, granulosa cells pulled away from the edge of the follicle but with spherical oocytes; G3, pyknotic nuclei, misshapen oocytes or vacuolation; Secondary/antral follicle: G1, intact spherical follicle with evenly distributed granulosa and theca cells, a small space and spherical oocytes; G2, intact theca cells, disrupted granulosa cells and spherical oocytes; G3, disruption and loss of granulosa and theca cells, pyknotic nuclei and missing oocytes.

### Enzyme-linked immunosorbent assays for E_2_ and FSH

Whole blood was collected from OT-transplanted mice 3, 7 and 21 days after transplantation. After centrifugation (3000 g for 5 min), an enzyme-linked immunosorbent assay (ELISA) was performed to analyze E_2_ and FSH (Cusabio, China) in sera. A microplate reader capable of measuring absorbance at 450 nm was used, and the concentrations of serum E_2_ and FSH were calculated. The intra- and inter assay precision coefficients of variation were < 15%. The minimum detectable dose of mouse E_2_ is typically less than 40 pg/ml, and FSH is less than 2.5 mIU/ml. A standard curve was created by reducing the data using computer software capable of generating a four-parameter logistic curve fit. E_2_ and FSH levels were calculated by the professional software ‘Curve Expert 1.4’ (Curve Expert, USA).

### Experiment II: further evaluation of the effects of resveratrol and exploration of the mechanisms

From follicle histological and endocrine function, we found that 45 mg/kg resveratrol was beneficial for the grafted ovary tissue. The mice were randomly divided into two groups: saline (*n* = 46) and 45 mg/kg resveratrol (*n* = 46).

### Apoptosis analysis

We observed follicle depletion in the early days of transplantation (day3 and day7). Therefore, we analyzed apoptosis on day 3 and day 7 between the two groups.

After deparaffinization and rehydration, sections were washed in PBS and treated with proteinase K (10 mg/ml, 37 °C, 30 min) in 10 mM Tris-HCl buffer. Then the sections were rinsed twice with PBS, they were incubated with 50 μl of terminal deoxynucleotidyl transferase-mediated dUTP nick-end labeling (TUNEL) reaction mixture for 1 h at 37 °C in a humidified chamber in the dark and rinsed with PBS twice. Negative controls were prepared by treatment with 1500 U/ml DNase I (Roche Applied Science) in 50 mM Tris–HCl (pH 7.5, including 1 mg/ml bovine serum albumin) for 10 min at room temperature to induce DNA strand breaks prior to the labeling procedures. Some OT specimens were used as negative controls by substituting terminal deoxynucleotidyl transferase with distilled water in the reaction mixture following the protocol. Mounting medium with 4′,6-diamidino-2-phenylindole (DAPI) was added (Vector Laboratories, USA), and the samples were examined under an inverted Zeiss AX10 microscope. An untreated TUNEL reaction mixture was used as a negative control, and a slide treated with 100 U/ml of DNase I was used as a positive control.

Six tissue sections from each group were obtained for analysis (*n* = 24). TUNEL-positive cells produced green fluorescence at excitation wavelengths. When 30% of the cells in one follicle were TUNEL positive, the follicle was regarded as apoptotic. DAPI produces fluorescence when bound to DNA.

### Immunohistochemistry

Ovaries were collected from OT-transplanted mice at 3 and 7 days. To explore the molecular mechanism of resveratrol, we tested relevant markers on the grafted ovary through immunohistochemical examination to validate the selected pathways. Sections were incubated for 1 h with primary antibodies against MDA (StressMarp, SMC-515, 1:800), SOD2 (Huabio, ET1701–54, 1:50), NF-κb (Huabio, R1309–9, 1:50), IL-6 (Huabio, R1412–2, 1:50), and SIRT1 (Huabio, ER1308–11, 1:50) at RT in a humid chamber, washed, and treated with EnVision goat anti-mouse IgG (Fuzhou Maixin, KIT-5006). A peroxidase substrate kit (SGK347011, Shanghai Gene) was used as a chromogen, and hematoxylin was used as a counterstain. Finally, six tissue sections from each group were obtained for analysis. The slides were examined under an inverted Zeiss AX10 microscope (Carl Zeiss, Germany). Brown coloring of the cytoplasm/nucleus of stromal cells, granulosa cells, or oocytes was defined as positive staining (any other coloring was considered negative staining).

### Gene expression analysis with real-time polymerase chain reaction

Ovaries were collected from OT-transplanted mice at 3 and 7 days, 8 mice per group were used for real-time quantitative PCR analysis (*n* = 32), and total RNA was isolated by using TRIzol (Invitrogen, Carlsbad, CA, USA). cDNA synthesis was performed with PrimeScriptTM RT Master Mix (Perfect Real Time). PCR analyses were performed with TB Green™ Premix Ex Taq™ II (TliRNaseH Plus) (TaKaRa, Code No. RR820A). The final PCR volume was 10 μl. The primer sequences used for real-time quantitative PCR are listed in Table 2.

**Table 2:** The primer sequences used for real-time quantitative PCR

Genes	Primers	Sequences (5′–3′)	Expected size (bp)
SIRT1	Sense	GCTGACGACTTCGACGACG	101
	Antisense	TCGGTCAACAGGAGGTTGTCT	
MDA	Sense	AGATGCCTTCAAACGGAGGAA	169
	Antisense	CAAGCTCAGAGTGGTGTGTCG	
SOD	Sense	AACCAGTTGTGTTGTCAGGAC	139
	Antisense	CCACCATGTTTCTTAGAGTGAGG	
IL-6	Sense	CCAAGAGGTGAGTGCTTCCC	117
	Antisense	CTGTTGTTCAGACTCTCTCCCT	
NF-κB	Sense	ATGGCAGACGATGATCCCTAC	111
	Antisense	TGTTGACAGTGGTATTTCTGGTG	
GAPDH	Sense	AGGTCGGTGTGAACGGATTTG	123
	Antisense	TGTAGACCATGTAGTTGAGGTCA	

The cycling conditions for the PCR were as follows: 95 °C for 30 s and 95 °C for 5 s for 40 cycles and 60 °C for 30 s for 40 cycles. Gene expression levels were evaluated using the delta-delta CT method and standardized to the GAPDH amplification levels.

### Western blot analysis

Ovaries were harvested on days 3 and 7 after transplantation and rapidly frozen in LN2, and six mice per group were used for western blot analysis (*n* = 24). Each ovary tissue sample was homogenized in ice-cold homogenization buffer containing protease inhibitors. Tissue homogenates were centrifuged at 5000×g for 5 min at 4 °C, and the supernatants were collected. The Bradford method was used for protein quantification. Proteins solubilized in 4 × Laemmli sample buffer were subjected to SDS-PAGE and then transferred onto nitrocellulose membranes. After the membranes were blocked with T-TBS for 1 h at room temperature, they were probed with various primary antibodies overnight at 4 °C [MDA (StressMarp, SMC-515, 1:1000), SOD2 (Huabio, ET1701–54, 1:1000), NF-κB (Huabio, R1309–9, 1:500), IL-6 (Huabio, R1412–2, 1:1000) and SIRT1 (Huabio, ER130811, 1:1000)]. After the membranes were washed with T-TBS, they were incubated with the corresponding secondary antibodies conjugated with horseradish peroxide for 1 h. Detection was conducted with an ECL kit (Millipore). Protein bands were scanned and analyzed with Quantity One Image Analysis Software (Bio-Rad, Hercules, CA, USA).

### Oocyte retrieval, in vitro maturation, IVF and in vitro culture

Twenty-one days after OT transplantation, 12 transplanted mice (6 mice/group) were hyperstimulated via intraperitoneal injection of 10 IU of pregnant mare’s serum gonadotrophin. After 48 h, cumulus–oocyte complexes (COCs) were obtained by ovary puncture and then matured in vitro in M16 medium (Sigma, USA) at 37 °C for 14 h. After in vitro maturation (IVM), nuclear maturation was examined. Extrusion of the first polar body was the maturation criterion and was scored under an inverted microscope (50× magnification). For sperm collection, mice were executed by cervical dislocation, and the abdominal skin was sterilized with 70% (v/v) alcohol. A 1.5-cm incision was made in midline abdominal wall, and the reproductive system was exposed. The head and tail of the epididymis and part of the vas deferens were cut together and then placed in culture medium in a glass dish. Fat tissue was removed under a microscope, and sperm inside the head of the epididymis and vas deferens were extruded to the cauda with ophthalmic forceps. Then, the heads of the epididymis and vas deferens were cut off. Sperm were released from the cauda of the epididymis and incubated for 1 h. The capacitation fluid containing sperm was absorbed and centrifuged at 1000 rpm for 4 min to collect sperm.

For fertilization, mature metaphase II (MII) oocytes were inseminated with sperm for 5 h, and the fertilized embryos obtained were washed with M16 medium and incubated at 37 °C for 24 h. Then, the fertilized embryos were placed in M16 medium (Global Media, LifeGlobal, Belgium) for further development. Fertilization was assessed by the formation of two cells 24 h after insemination. The cleavage and blastocyst rates were calculated.

### Statistical analysis

The Statistical Package for the Social Sciences version 24.0 software (SPSS, Inc., USA) and GraphPad Prism 5.0 (GraphPad Software, USA) were used for statistical analysis. Unless otherwise indicated, the results are shown as the mean ± SD. Data were analyzed by Student’s t-test (TUNEL, MII oocytes, fertilization, cleavage and blastocyst formation), one-way analysis of variance (serum FSH and E_2_, qRT-PCR, and western blot), and χ2 tests (the proportions of G1 follicles). *P* < 0.05 was considered significant.

## Results

### Evaluation of the best concentration of resveratrol (experiment I)

#### Follicle classification and morphological analysis

**Fig. 1:**
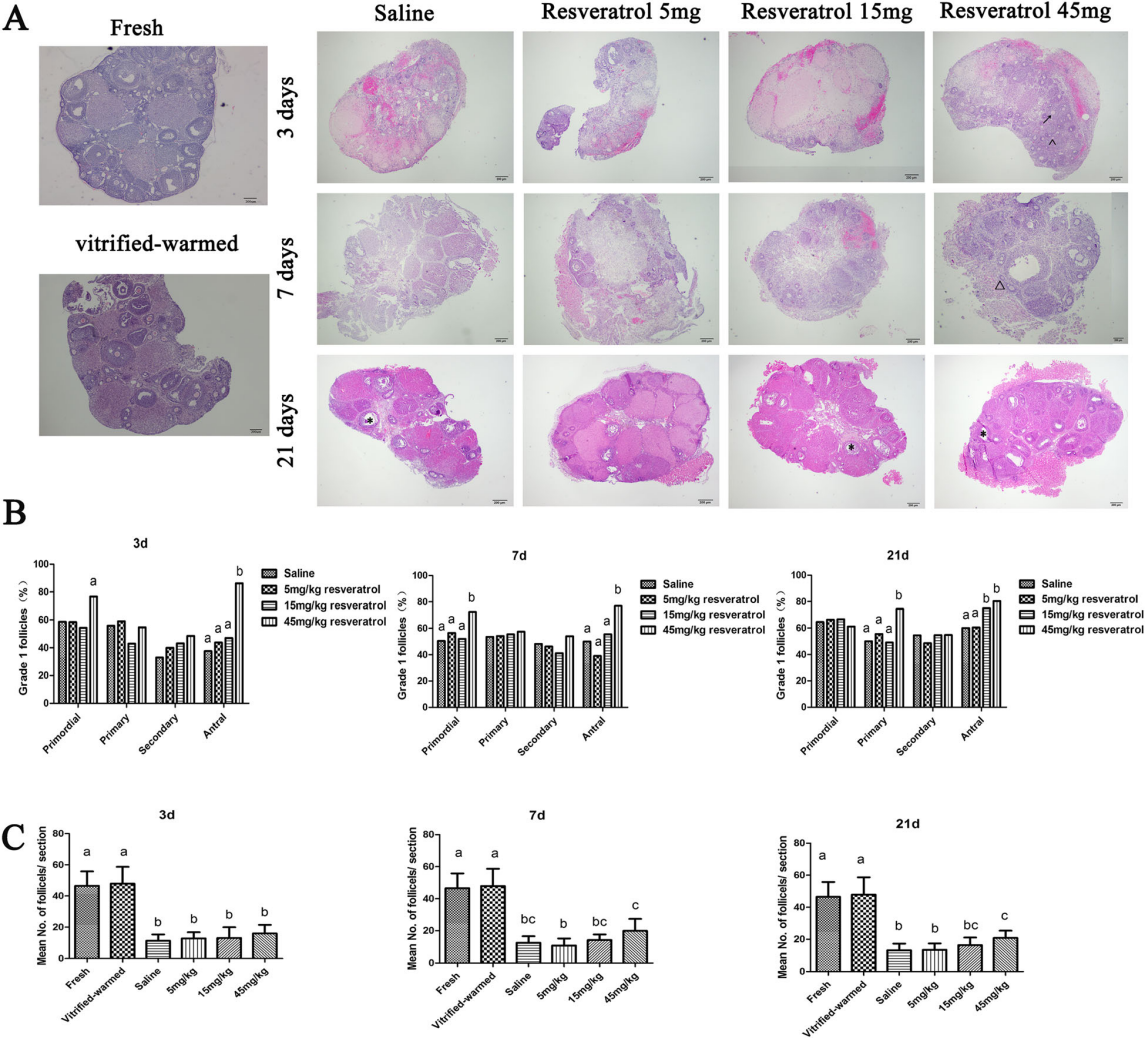
Analysis of recovered ovarian tissue after ovarian transplantation. **a**: Representative images of hematoxylin and eosin-stained fresh, vitrified and transplanted ovarian tissues. The magnification is 40×, and the scale bar is 200 μm. △, primary follicles; ↑, secondary follicle; ^, primordial follicles; *, antral follicles. **b**: The proportions of G1 follicles in mouse ovaries on days 3, 7 and 21. Data were analyzed by the χ^2^ test, and different letters indicate statistically significant differences (*P* < 0.05) C: The mean G1 follicle number per section of the mouse ovary on days 3, 7 and 21. Data were analyzed by one-way analysis of variance. Graphs show the mean ± SD, and different letters indicate statistically significant differences (*P* < 0.05)

The frozen-thawed OT appeared well preserved; 75% of the follicles were primordial, primary and secondary; and the stromal cells showed an intact shape. However, the proportions of Grade 1 antral follicles decreased, and the differences were highly significant (fresh: 67.6%; cryopreservation-warmed: 40%). On morphological analysis, the antral follicles showed atresia, and the surrounding granular cells were loose. On day 3 after OT transplantation, the transplanted ovaries could be easily detached from the kidneys, although many red blood cells were present around the kidney capsule (Supplementary, Fig. S). However, adhesion between the grafts and the kidneys was enhanced in a time-dependent manner. On day 21 after transplantation, the blood vessels in the transplanted OT increased substantially. The quantity and quality of ovarian follicles at all stages deteriorated after transplantation compared with those of fresh OT and cryopreservation-warmed OT, and oocyte shrinkage and a collapsed follicular structure were observed (Fig. 1a). The proportion of Grade 1 primordial follicles significantly increased in the 45 mg/kg resveratrol group compared with the saline and other resveratrol-treated groups on days 3 and 7, which was consistent with observations of antral follicles (Fig. 1b). Moreover, on day 21, resveratrol–treated (45 mg/kg) ovarian grafts showed increased G1 follicle ratios in the developmental stages (primary and antral follicles) compared with grafts in the other three groups. Figure 1c shows the mean number of total G1 follicles in each stage per section before and after cryopreservation and transplantation in the four different groups on days 3, 7 and 21. A 50% reduction in the mean G1 ovarian follicle number was observed with autotransplantation in the four transplantation groups compared with the fresh control and vitrification-warmed control groups. However, the 45 mg/kg resveratrol group showed a significantly higher number of G1 follicles than the 5 mg/kg group at 7 days and the saline and 5 mg/kg groups at 21 days (Fig. 1c).

### Serum E_2_ and FSH levels

**Fig. 2:**
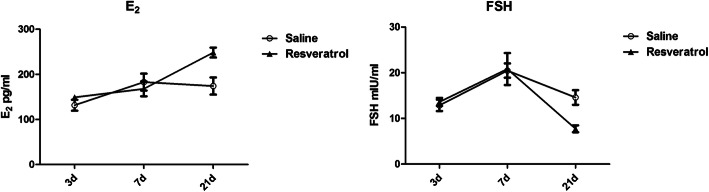
Serum E_2_ and FSH levels in autotransplanted mice. Serum E_2_ and FSH levels were detected by enzyme-linked immunosorbent assay on days 3, 7 and 21. Data were analyzed by analysis of variance. Graphs show the mean ± SD, and different letters indicate statistically significant differences (*P* < 0.05)

The serum concentrations of FSH and E_2_ at 3 and 7 days after transplantation were not different between the two groups (Fig. 2). Furthermore, we found that FSH increased on day 7 after transplantation and was higher than that on day 3, although no difference was found between the two groups. However, on day 21 after transplantation, we found that the serum FSH level was significantly decreased in the resveratrol group compared with the saline groups (*P* < 0.05). The serum E_2_ level was significantly increased in the resveratrol group, and the E_2_ level was highest in this group; these changes were significant.

### The effects of resveratrol and the mechanisms on autotransplantation of frozen-thawed mouse ovarian tissue (experiment II)

#### Apoptosis analysis

**Fig. 3:**
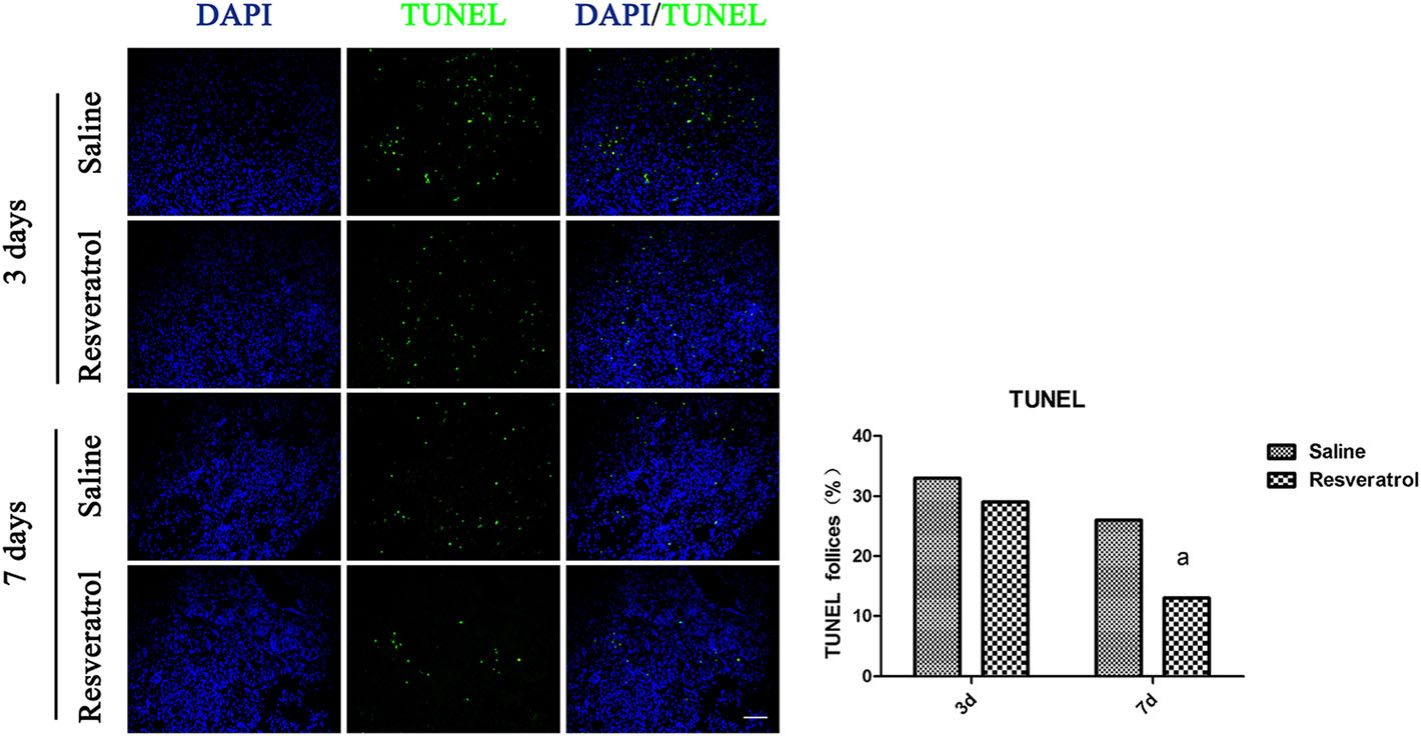
Terminal deoxynucleotide transferase–mediated dUTP nick-end labeling staining showing apoptotic follicles (green, TUNEL) and cell nuclei (blue, DAPI) between the two groups on days 3 and 7 after ovarian transplantation. The letters indicate statistically significant differences (*P* < 0.05) in the apoptotic follicle ratio between the two groups on day 7 after ovarian transplantation. The magnification is 200×, and the scale bar is 50 μm

TUNEL-positive follicles were detected in the two groups on days 3 and 7 (Fig. 3). Three days after transplantation, the two groups showed an approximately 30% apoptotic follicle ratio. The apoptotic follicle ratio decreased in the 7-day group, and 13% of positive staining signals were detected in the resveratrol group. The apoptotic follicle ratio was significantly decreased in the 45 mg/kg resveratrol group compared with the saline group.

#### Immunohistochemistry, gene expression and western blot analysis

**Fig. 4:**
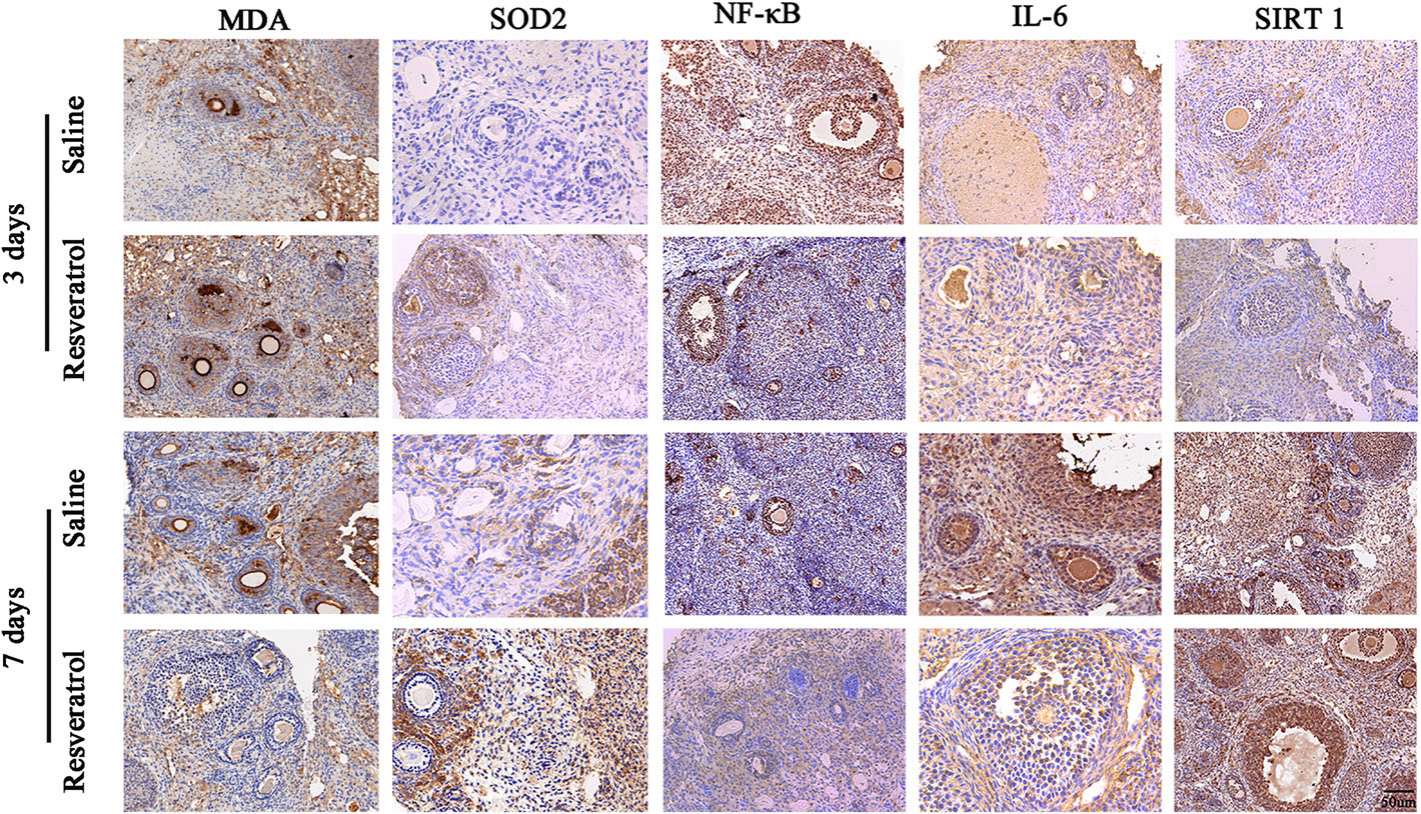
Immunohistochemical staining of ovarian tissue (OT) for MDA, SOD2, NF-κB, IL-6 and SIRT1. The magnification is 200×, and the scale bar is 50 μm

**Fig. 5:**
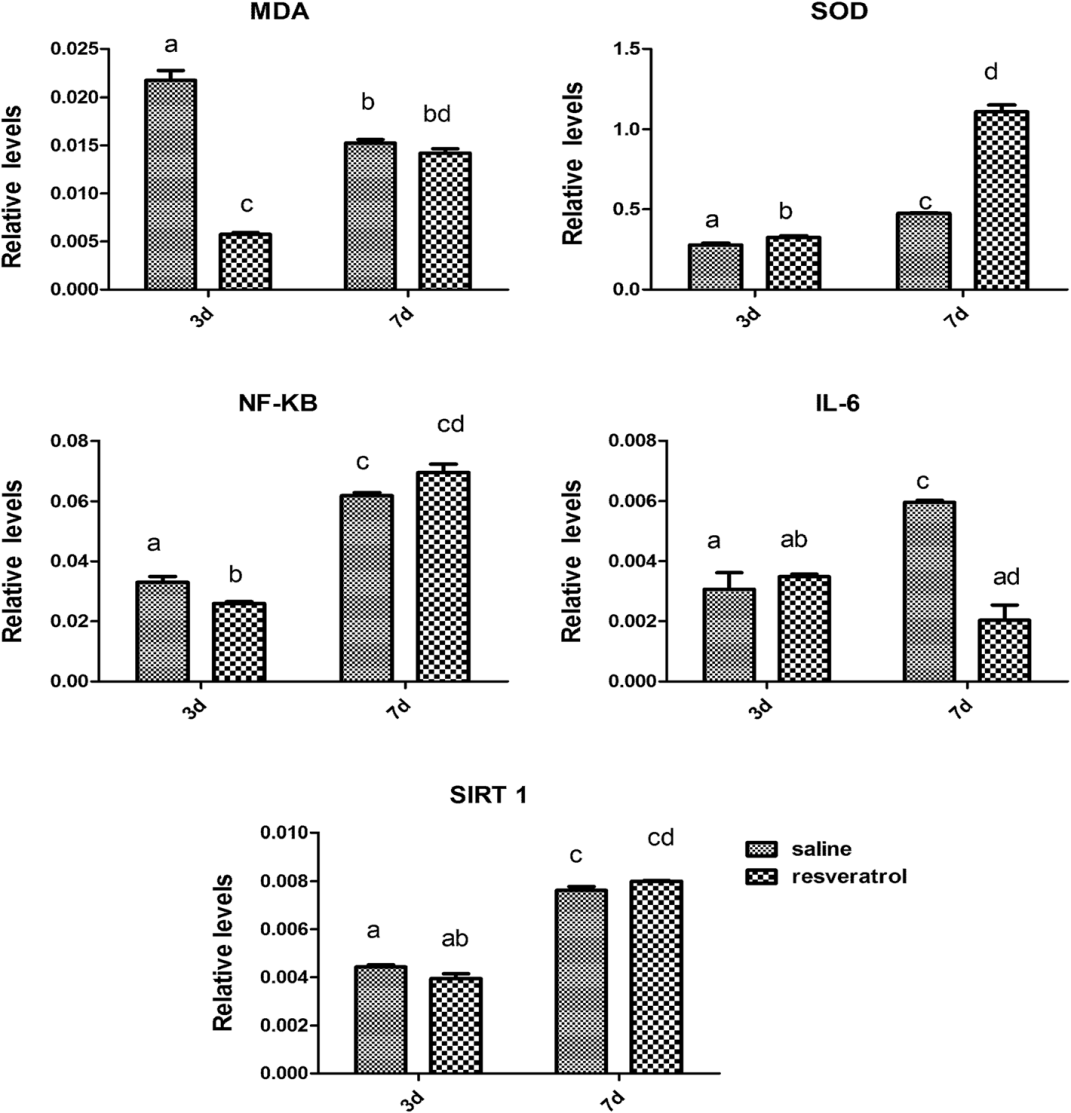
The relative levels of MDA, SOD, NF-κB, IL-6, and SIRT 1 mRNA by real-time PCR in ovarian tissue 3 days and 7 days after auto-transplantation. Data represent the mean ± SD. The different superscript letters represent significant differences between the two groups at different times

Figure 4 shows a representative immunohistochemical image of MDA-, SOD2-, NF-κB-, IL-6-, and SIRT1-positive areas in the ovary at 3 and 7 days after transplantation. MDA is a marker of oxidative stress and is mainly expressed in the nuclei of oocytes and other various cells in OT. SOD2, a subtype of SOD, is mainly expressed in the oocyte cytoplasm of various cells in OT. NF-κB is a nuclear transcription factor expressed in the oocyte nucleus, granule cells and basal interstitial cells. IL-6 is widely expressed in the nuclei and cytoplasm of various cells in the ovary. SIRT1 is mainly expressed in the oocyte nucleus, granule cells and basal interstitial cells. Real-time PCR demonstrated that the levels of MDA and NF-κB were significantly lower in the 45 mg/kg resveratrol group than in the saline group at day 3 (Fig. 5). IL-6, a proinflammatory cytokine, was lower in the 45 mg/kg than that in the saline group on day 7. Furthermore, a significant increase in SOD levels was observed on days 3 and 7 compared in the 45 mg/kg with those in the saline group. However, no difference in SIRT 1 was observed between auto-transplanted OT treated with 45 mg/kg resveratrol and OT in the saline group.

**Fig. 6:**
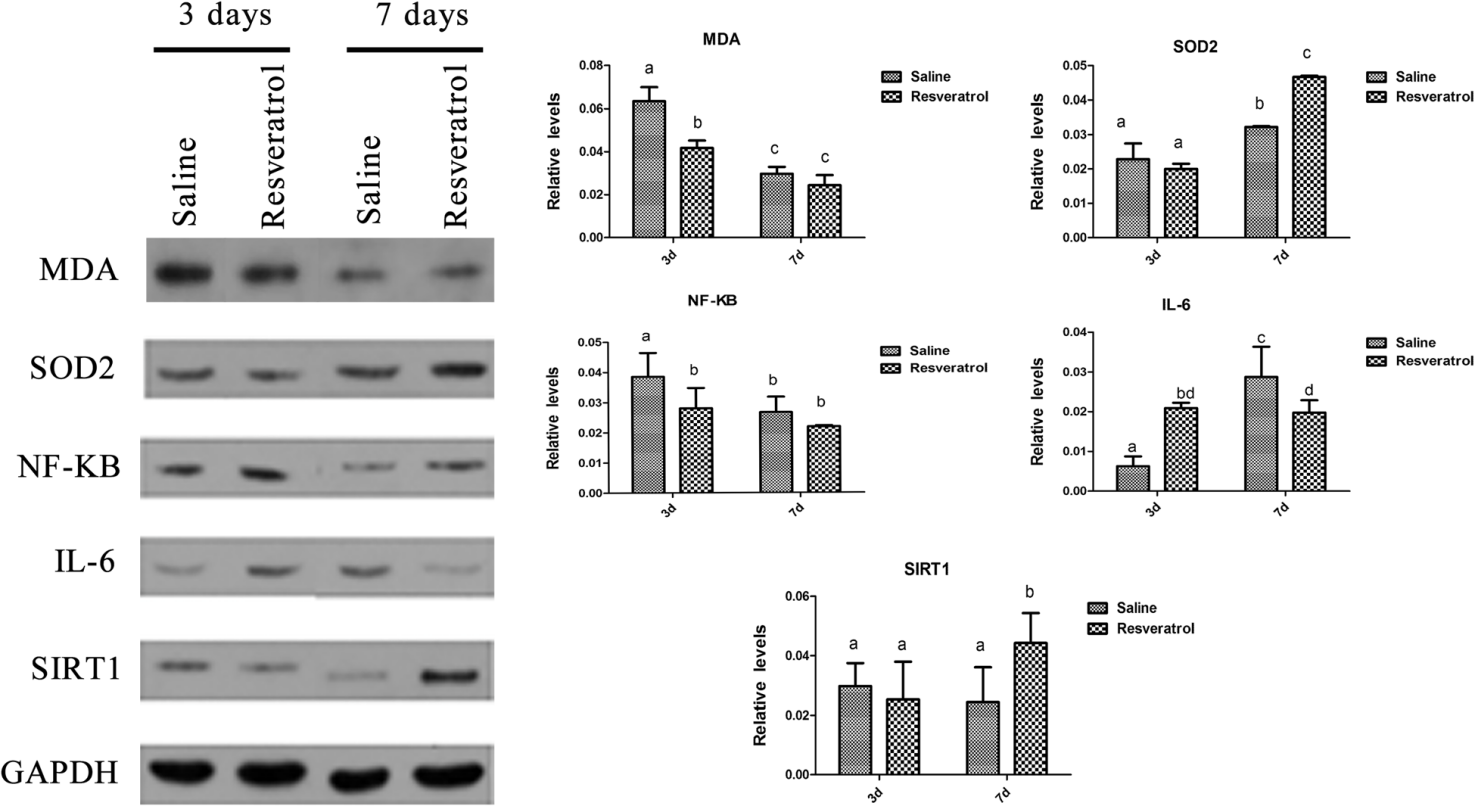
The relative levels of MDA, SOD2, NF-κB, IL-6 and SIRT1. For western blotting, GAPDH was used as a loading control in ovarian tissues 3 days and 7 days after auto-transplantation

Western blot analysis revealed that the levels of SOD2 and SIRT1 were significantly higher in the resveratrol group than in the saline group on day 7 (Fig. 6). Furthermore, MDA and NF-κB levels were lower in the resveratrol group than those in the saline group on day 3. Likewise, IL-6 was lower in the resveratrol group than that in the saline groups on day 7. These results are basically consistent with the qRT-PCR results.

### IVM, IVF, and in vitro embryonic development derived from autotransplanted ovaries

**Fig. 7:**
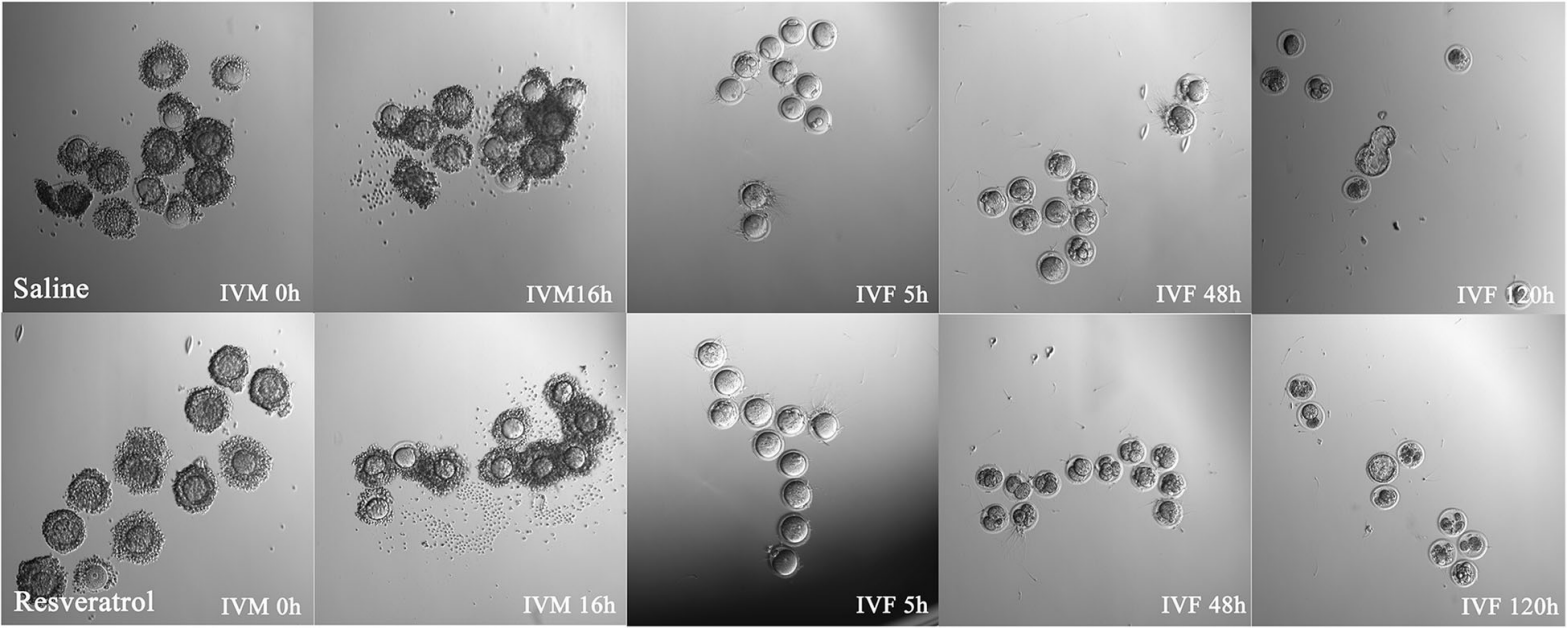
The embryonic development of mouse oocytes retrieved from ovarian tissue grafts 21 days after transplantation. (IVM and IVF oocytes; the magnification was × 100)

Figure 7 and Table 3 show data for the embryonic development of oocytes retrieved from OT grafts 21 days after transplantation. The mean number of retrieved oocytes and fertilization and cleavage were significantly increased in the resveratrol group compared with the saline group (*P* < 0.05) but not the MII group. Although blastocysts were cultured after insemination in the two groups, no significant difference was noted. The number of blastocysts was insufficient, and most embryos eventually degenerated.

**Table 3:** The embryonic development of mouse oocytes retrieved from ovarian tissue grafts 21 days after transplantation. MII, Metaphase II (Mean ± SD)

Group	No. of retrieved oocytes	No. of MII oocytes	No. of fertilized oocytes	No. of cleaved oocytes	No. of blastocysts
Saline (from 8 ovaries)	14.88 ± 2.75	9.63 ± 2.13	5.88 ± 1.81	4.50 ± 1.51	0.5 ± 0.76
Resveratrol (from 8 ovaries)	20.63 ± 5.07	11.13 ± 3.40	9.88 ± 3.48	8.50 ± 3.02	0.5 ± 0.54
t	−2.82	−1.06	−2.89	−3.35	0.00
p	0.01	0.31	0.01	0.01	1.00

*t* Student’s t-test, *p* value

## Discussion

Hypoxia and ischemia before neovascularization are major concerns in OT transplantation because they cause extensive follicle loss [31]. Increasing the lifespan and quality of grafts, which may result in improved pregnancy outcomes for cancer patients, has been a major challenge in recent years [14]. A microdialysis probe inserted in grafted OT showed long progression from anaerobic to aerobic metabolism and a protracted period of ROS generation [32]. Oxidative stress was observed relatively late, after the most critical period of follicle loss, and lasted until the tissue vasculature stabilized [32].

In the present study, our results show that the proportion of G1 follicles was significantly higher, and only 13% of TUNEL positive staining signals were detected in the 45 mg/kg resveratrol group; additionally, in the same group, recovered at day 21. IHC, PCR and WB showed that SOD2 and SIRT1 were significantly higher, while MDA and NF-κB were lower than those in the saline group on day 3, and IL-6 was lower than that in the saline group on day 7. Based on the results, resveratrol was believed to prevent ovarian follicle damage and restore ovarian function promptly. In addition, we speculated that signaling pathway involved oxidative stress and anti-inflammatory could play an important role after transplantation.

A substantial reduction in the percentage of morphologically normal antral follicles was noted. Because antral follicles are very large and surrounded by multiple layers of cuboidal granulosa cells, they could not tolerate the freezing damage and maintain their structural integrity during vitrification [11]. Nevertheless, preantral follicles can maintain their follicular structure and survive the transplantation procedure [10, 33]. Vitrification is a kind of technology that is easy to perform for the simple and quick preservation of ovaries. Moreover, the conventional slow-freezing is commonly regarded to inevitably introduce ‘two factors’ injuries to the cells, i.e. the injuries caused by intra-cellular ice formation and the elevated concentration of the residual solution [27, 34]. Vitrification has been shown to overcome these problems. Therefore, vitrification is an effective method for fertility preservation as previous research has shown that antral follicles damaged in cryopreservation have no effect on transplantation. Post-transplantation damage is the main cause of ovarian injury during cryopreservation and transplantation [35].

Previous reports revealed that granulosa cells and oocytes, particularly in developing follicles, undergo apoptosis as a result of ischemia-reperfusion injury induced by free radicals and lipid peroxidation on the first day of transplantation [36]. This process can lead to degeneration and atresia of follicles and disruption of folliculogenesis and oogenesis. In this study, we tested different concentrations of resveratrol to demonstrate its effect (5 mg/kg, 15 mg/kg and 45 mg/kg) and found that, In the 45 mg/kg resveratrol group, the G1 rate of primordial follicles on day 3 was significantly higher than that in the saline group, and the same results were observed at 7 days. Based on these results, we used this concentration for Experiment II. Moreover, the ratio of TUNEL-positive follicles decreased in the resveratrol group 7 days after transplantation. The primordial follicles shifted to secondary follicles and antral follicle on day 21 after transplantation. This trend suggests that resveratrol may improve the quality of follicles by reducing follicle apoptosis after transplantation. The mean number of follicles in grafted OT decreased compared with that in cryopreservation-warmed OT (24.25 ± 6.51).

FSH and E_2_ levels are widely used to evaluate ovarian reserve. The FSH levels on day 3 were substantially higher than the normal range (lower than 10 ng/ml) due to the absence of negative feedback from the ovary during the period between ovariectomy and OT survival [37]. However, after OT ischemia-reperfusion and survival, ovarian function was evaluated by detecting FSH and E_2_ levels, which recovered on day 21, indicating that the transplanted OTs functionally recovered after 21 days in the mouse model. Restoration of ovarian function post-transplantation was also indicated by the presence of antral follicles and corpus luteum in the OT.

To our knowledge, grafted OT undergoes a protracted period of ROS generation, which can cause oocyte, granulosa cell and membrane lipid damage or apoptosis [32]. Resveratrol is a strong antioxidant, and our results revealed that resveratrol can improve the quality of ovarian tissue after autotransplantation. To explore the mechanisms of resveratrol, we examined several factors related to oxidative stress (SIRT1, MDA and SOD2) and inflammation (NF-κB and IL-6) using immunohistochemistry. We found that these five specific factors were all observed in different cell of the grafts by IHC.

Immunohistochemistry, Quantitative real-time PCR and western blots revealed the potential mechanisms of resveratrol in our study. Resveratrol could decrease the level of MDA by increasing SOD to reduce oxidative stress [38]. Another explanation for this mechanism is that resveratrol inhibits NF-κB and interfere with NF-κB signal transduction on day 3 and results in reduced release of proinflammatory cytokines, such as IL-6, 7 days after transplantation [39]. NF-κB [40] is a nuclear transcription factor that mediates intracellular signal transduction. SIRT1 regulates the activation or inhibition of multiple target proteins. Both in vivo and in vitro, the antioxidant and anti-inflammatory actions of resveratrol have been reported to be mediated by SIRT1 activation [41]. However, no significant differences were observed in the relative level of SIRT1 as shown by qRT-PCR, whereas a significant increase was observed at the protein level. SIRT1 is a highly conserved NAD + -dependent deacetylase that plays a cellular protective role by deacetylating substrate proteins to resist various stressors and repair gene mutations [42]. We speculated that after transcription, the mRNA must pass through the nuclear membrane to be translated into a protein [42]. Resveratrol might promote the translation of SIRT1.

In the present study, we found that antral follicles could be observed 21 days after transplantation, and that the rate of oocyte induction by puncture was significantly lower than that of normal ovaries from controlled ovarian hyperstimulation (COH) [43]. A significant increase in the number of MII follicles in the resveratrol group compared with the saline control group was observed. After IVF, the fertilization and cleavage rates in the resveratrol group were higher than those in the saline group. Resveratrol increased the cleavage rate, but no difference in the number of blastocysts was found between the two groups. Due to the small number of cultured blastocysts, further testing the quality of the embryos was difficult, and most embryos eventually degenerated. In vivo transplantation may be an ideal method, and more studies are needed in the future.

## Conclusion

This study demonstrated that resveratrol can improve the efficacy of autotransplantation of frozen-thawed mouse OT through anti-inflammatory and antioxidative mechanisms, which is consistent with the increased follicle survival rate and reduced follicle apoptosis. However, this study did not assess metabolic parameters. We tested the effect of resveratrol on grafted tissue, which is a preliminary experiment; therefore, further studies are necessary to elucidate the mechanisms of resveratrol in minimizing ischemia-reperfusion injury in the ovaries of other domestic animals and in humans.

## Supplementary Information

**Additional file 1: Figure S** Representative images of mouse ovarian tissue grafts according to the duration of transplantation. The arrow indicate blood vessel.

## Abbreviations

OT: Ovarian tissue
OTCT: Ovarian tissue cryopreservation and transplantation
ROS: Reactive oxygen species
IP: Intraperitoneal
ES: Equilibration solution
VS: Vitrification solution
DPBS: Dulbecco’s phosphate-buffered saline
TUNEL: Terminal deoxynucleotidyl transferase-mediated dUTP nick-end labeling
DAPI: 4′,6-diamidino-2-phenylindole
ELISA: Enzyme-linked immunosorbent assay
PCR: Polymerase chain reaction
RBCs: Red blood cells
