# Supplementary material for: Effect of resveratrol on mouse ovarian vitrification and transplantation
Source: Reprod Biol Endocrinol. 2021 Apr 9;19:54. doi: 10.1186/s12958-021-00735-y (PMC8033708; doi:10.1186/s12958-021-00735-y)
Supplement: Supplementary file 1 — Additional file 1: Figure S Representative images of mouse ovarian tissue grafts according to the duration of transplantation. The arrow indicate blood vessel. [file 12958_2021_735_MOESM1_ESM.docx]

Fig. S Representative images of mouse ovarian tissue grafts according to the duration of transplantation. The arrow indicate blood vessel.
